# Heat Stress Increases Movement Jerk During Physical Exertion

**DOI:** 10.3389/fphys.2021.748981

**Published:** 2021-10-25

**Authors:** David Hostler, Jacqueline Schwob, Zachary J. Schlader, Lora Cavuoto

**Affiliations:** ^1^Department of Exercise and Nutrition Sciences, Center for Research and Education in Special Environments, University at Buffalo, Buffalo, NY, United States; ^2^Department of Kinesiology, Indiana University, Bloomington, IN, United States; ^3^Department of Industrial and Systems Engineering, University at Buffalo, Buffalo, NY, United States

**Keywords:** heat stress, jerk, acceleration, occupation, wearable sensor

## Abstract

**Objective:** Movement efficiency can be quantified during physical tasks by measuring the rate of change of acceleration (jerk). Jerk captures the smoothness of a motion and has been used to quantify movement for upper extremity and torso-based tasks. We collected triaxial accelerometer data during four physical tasks commonly performed in the work place to determine if jerk increases with physiologic strain.

**Methods:** Participants completed a circuit of activities that mimicked the demands of manual labor in hot (40°C) and temperate (18°C) conditions. The circuit included walking on a treadmill carrying a load on the shoulder, lifting objects from the floor to the table, using a dead blow to strike the end of a heavy steel beam, and a kneeling rope pull. After the 9 min circuit, the participant had a standing rest for 1 min before repeating the circuit 3 additional times. Participants were instrumented with four 3-axis accelerometers (Actigraph wGT3X) secured to the torso, wrist, and upper arm.

**Results:** There were 20 trials in the hot condition and 12 trials in the temperate condition. Heart rate and core body temperature increased during both protocols (*p* < 0.001). Measures of jerk varied by accelerometer location and activity. During treadmill walking, the wrist, torso, arm accelerometers measured higher jerk during the fourth circuit in the hot condition. During the lifting task, mean jerk increased in the hot condition in all accelerometers. Max jerk increased in the temperate condition in the arm accelerometer and jerk cost increased in the hot condition in the torso and arm accelerometers.

**Conclusions:** Forty minutes of paced work performed in the heat resulted in increased acceleration and jerk in accelerometers placed on the torso, arm, and wrist. The accelerometers most consistently reporting these changes were task specific and suggest that a limited number of worn sensors could identify the onset of fatigue and increased injury risk.

## Introduction

Public safety, occupational, and military agencies are interested in real-time physiologic monitoring of vital signs, such as heart rate and core temperature (Coca et al., 2010; Friedl, 2018; Morrissey et al., 2021). Despite considerable research investment, such monitors have not been widely deployed in the workforce. This, in part, is due to the complexity of human physiology and the need to understand the normal responses of each individual wearing the monitor. For example, younger individuals can safely tolerate higher heart rates during exertion compared to older individuals. Even within a given age group, fit individuals would tolerate higher heart rates than unfit individuals but also work at lower heart rates at any given submaximal intensity. Therefore, it is nearly impossible to define a single algorithm based on heart rate that triggers a reliable advisory or alarm alerting the end user of excessive physiologic load and increased risk of injury during exertion.

Movement efficiency can be quantified during physical tasks based on the measurement of jerk, which is defined as the rate of change of acceleration. Jerk captures the smoothness of a motion and has been used in the occupational ergonomics, rehabilitation, and motor control domains to quantify movement for upper extremity and trunk-based tasks (Hogan et al., 1987; Chang et al., 2005; Cote et al., 2005). A lower jerk is indicative of a smoother movement pattern, while increased jerk (i.e., large changes in acceleration) results in large forces on the body. Movements high in jerk should be avoided as they may be associated with an increased risk of musculoskeletal injury (Danz and Ayoub, 1992; Waters et al., 1993; Lavender et al., 2012).

Previous work from our group has shown that movement becomes less fluid during prolonged exertion and a characteristic jerk is seen in accelerometer data collected during exertion (Ghesmaty Sangachin and Cavuoto, 2016; Sedighi Maman et al., 2017). Presumably, this sign has a temporal relationship to perceived and/or objectively measured fatigue and could be used in a wide range of individuals and settings. In this pilot study, we collected triaxial accelerometer data during four physical tasks (walking, lifting, striking, pulling) performed in temperate and hot conditions to determine if jerk increases with increasing physiologic strain.

## Materials and Methods

The data were collected from two sub studies from a larger project. While the studies differed in data collected before and after exertion, both studies utilized identical procedures with regards to the exercise protocol and accelerometer data collection. In one study, subjects completed exercise in temperate [18.4 ± 1.0°C, 38.0% relative humidity (RH)] and hot conditions (40.1 ± 1.0°C, 26.5% RH), while subjects in the second study only completed the hot condition (40.8 ± 0.7°C, 24.4% RH). Both studies were approved by the University at Buffalo Institutional Review Board.

### Population and Recruitment

Nineteen subjects (eleven males) provided written informed consent (Table 1). Three subjects participated in both studies. Subjects were recruited from the university population. Exclusion criteria included metabolic, neurologic, respiratory, or cardiac diseases, a previous abdominal surgery, and the use of medications known to influence responses to exertion or thermoregulation. Females were screened for pregnancy at each visit and completed their exercise trials during the first 10 days following self-identified menstruation.

**TABLE 1:** Morphometrics of the subject pool.

	Age (y)	Height (cm)	Mass (kg)	BMI
All (*n* = 19)	23.8 ± 3.6	168 ± 10	69.0 ± 12.4	24.2 ± 3.1
Male (*n* = 11)	23.8 ± 4.0	175 ± 7	76.8 ± 9.0	25.0 ± 2.1
Female (*n* = 8)	23.9 ± 3.3	159 ± 5	58.3 ± 7.5	23.3 ± 4.0

*Data presented as mean ± SD.*

### Instrumentation

Core body temperature was measured using a telemetry capsule (CorTemp, HQ, Inc.). Height was measured with a stadiometer and mass with a digital scale. Temperature data loggers (iButton, Thermochron) were applied to the clavicular head of the pectoralis major, the triceps brachii, anterior of the quadriceps muscles, and the gastrocnemius on the right side of the body to measure skin temperature. Skin temperature was sampled every 60 s and weighted mean skin temperature (MST) calculated by averaging the values in a 4-min window using the equation: MST = 0.3 (chest + arm) + 0.2 (quad + calf) (Ramanathan, 1964). Body and limb acceleration were recorded by accelerometers at a sampling rate of 100 Hz (ActiGraph wGT3X-BT) placed over the wrist, torso, and triceps brachii on the non-dominant side of the body. Accelerometers measured accelerations in the local x, y, and z planes of each location.

### Testing Protocol

Subjects arrived at the laboratory having abstained from alcohol, caffeine, exercise, and nicotine for 12 h and food for 2 h. The telemetry capsule was administered 1 h prior to beginning exercise. Next, subjects provided a urine sample to verify euhydration defined as a urine specific gravity of less than 1.025. Baseline nude mass and supine resting vital signs (heart rate, blood pressure, and respiratory rate) were collected prior to instrumentation.

**FIGURE 1:**
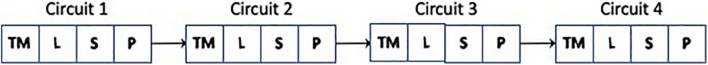
Each circuit of the protocol included treadmill walking (TM) with a load on one should, a lifting task L, a striking task (S), and a pulling task (P). Circuits were separated by 1 min of standing rest (arrow).

The exercise trial was conducted in an environmental chamber. The hot condition was set to 41°C and 20% relative humidity while the temperate condition was set to 18°C and 20% relative humidity. Baseline measurements of heart rate and core temperature were collected immediately upon entering the environmental chamber. The exercise circuit consisted of four exercises (Figure 1). Subjects were allowed to practice the movements at their screening visit. The circuit was completed a total of four times during the trial. The first task involved walking on a treadmill at 5.6 kph at 0% grade carrying a 15.2 m length of bundled 4.4 cm fire hose weighing 8.7 kg over one shoulder for 3 min. Next, a lifting task was performed by moving one of five objects (three weighing 4.1, 5.4, and 6.8 kg and two weighing 21.1 kg) from a 73 cm platform to the floor and returning them to the platform every 12 s for 2 min. The third exercise in the circuit required the subject to use a 4.1 kg dead blow to strike the end of a 72 kg I-beam along a track. The subject hit the beam every 10 s for 2 min. The final exercise required the subject to pull a 4.4 cm wide hose looped around a pivot point while in a kneeling position. The hose was pulled continuously for two 45 s intervals separated by a 15 s rest. Finally, a 60 s standing rest took place before the start of the next circuit. Subjects repeated the circuit four times, for a total of 40 min of exertion. Heart rate, core temperature, and mean skin temperature were collected throughout the protocol. The exercise trial was terminated if the subject’s heart rate exceeded their age-predicted maximum (i.e., 220–age) for two consecutive measures, core temperature exceeded 39.5°C, or if the subject requested to stop. The subjects were reweighed nude after exiting the environmental chamber.

### Data Reduction

The raw signal accelerometer signals for each accelerometer location were bandpass filtered from 0.1–10 Hz. The jerk signals were calculated from the filtered accelerometer signals as $J\,e\,r\,k_{k,i}=\frac{a_{k,i}-a_{k,i-1}}{△\,t},$ for *k* = {*x*,*y*,*z*}, *i* = 2:*T*, Δ*t* = 1/100 s, and *T* is the length of the trial. The magnitude of the jerk signal was then calculated. Jerk magnitude vectors for each accelerometer location were segmented by circuit (1–4) and task (walking, lifting, striking, pulling). For the first and last circuits (1 and 4) for each task and accelerometer location, three features were extracted: mean jerk, maximum jerk, and jerk cost ($Jerk_{c\,o\,s\,t}=\sum_{i=1}^{t}J_{i}^{2}△t)$. These two circuits were selected to represent the least and most fatigued time points. Data from one session each for two participants had to be excluded due to sensor malfunction. This impacted all four sensors for one participant and only the arm sensor for the second.

### Statistical Analyses

Changes in heart rate, core body temperature, and mean skin temperature were measured before and at the end of each circuit and examined over time and between groups *via* a two-way mixed model ANOVA with a repeated factor of circuit and a between subject factor of environmental condition. *Post hoc* comparisons were performed with Sidaks multiple comparisons test. Jerk measures were log transformed and compared with the same ANOVA and *post hoc* parameters but only the first and fourth circuits were compared. Changes in body mass during exertion were compared by *t*-test. All tests were performed using GraphPad Prism version 9.0.0 for Mac, GraphPad Software, San Diego, CA, United States.

## Results

**FIGURE 2:**
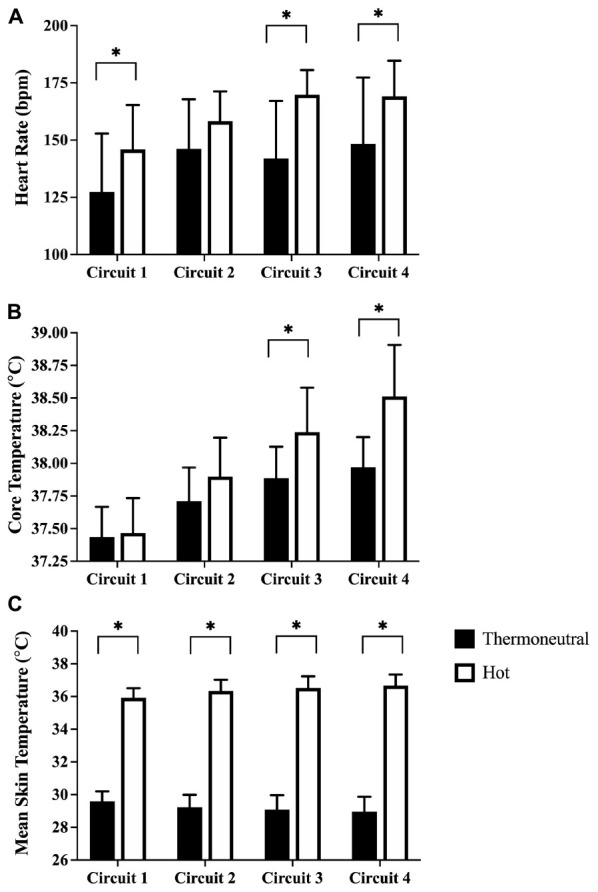
Heart rate (top), core body temperature (middle), and mean skin temperature (bottom) in the temperate and hot conditions at the end of each round of work. Data presented as mean ± SD. ^∗^Values are different (*p* < 0.05) at the beginning and end of the round.

There were 20 trials in the hot condition and 12 trials in the temperate condition. Subjects lost 0.33 ± 0.16 kg of body mass during activities performed in the temperate condition and 0.79 ± 0.20 kg in the hot condition (*p* < 0.001). Heart rate and core body temperature increased during both protocols (*p* < 0.001). Heart rate was higher in the hot condition compared to the temperate condition at the end of the first, third, and fourth circuits (Figure 2A). Core temperature was higher in the hot temperature at the end of the third and fourth circuits while mean skin temperature was higher in the hot condition at the end of every circuit (Figures 2B,C).

During treadmill walking, mean jerk and jerk cost increased in the wrist, torso, and arm accelerometers in the hot condition. Maximum jerk cost increased in the arm and torso accelerometers in the hot condition and in the torso accelerometer in the temperate condition (Table 2). Jerk cost was higher during circuit 4 of the hot condition compared to circuit 4 of the temperate condition.

**TABLE 2:** Jerk measures measured during treadmill walking with a load on one shoulder during the first and fourth bout of work.

Treadmill Walking						

Mean jerk (units: g/s)						

	Wrist		Torso		Arm	
			
	Hot	Temperate	Hot	Temperate	Hot	Temperate
Circuit 1	5.387 ± 1.455	5.218 ± 0.812	6.159 ± 0.966	5.937 ± 0.942	6.118 ± 1.109	5.852 ± 0.825
Circuit 4	6.119 ± 1.558*	5.468 ± 0.989	6.841 ± 1.136*	6.387 ± 1.030	6.585 ± 1.189*	6.021 ± 1.159

**Maximum jerk (units: g/s)**						

	**Wrist**		**Torso**		**Arm**	
			
	**Hot**	**Temperate**	**Hot**	**Temperate**	**Hot**	**Temperate**

Circuit 1	23.29 ± 8.55	25.11 ± 10.62	12.67 ± 2.60	11.52 ± 1.70	16.52 ± 3.05	15.64 ± 2.67
Circuit 4	29.45 ± 11.57	25.69 ± 10.73	16.07 ± 4.12*	13.93 ± 2.52*	21.72 ± 8.50*	18.54 ± 5.23

**Jerk cost (units: g^2^/s)**						

	**Wrist**		**Torso**		**Arm**	
			
	**Hot**	**Temperate**	**Hot**	**Temperate**	**Hot**	**Temperate**

Circuit 1	6,725 ± 4,039	5,976 ± 1,901	7,697 ± 2,638	7,172 ± 2,340	7,735 ± 2,962	7,024 ± 2,034
Circuit 4	8,907 ± 4,810*	6,865 ± 2,821	9,701 ± 3,414*	8,528 ± 2,816 +	9,197 ± 3,455*	7,705 ± 3,233

*Data presented as mean ± SD.*

*Circuit 4 different from circuit 1 in that condition (*p* < 0.05).

*+Circuit 4 in the hot condition is different from circuit 4 in the temperate condition.*

During the lifting task, there was an effect of time in the torso and wrist accelerometers for maximum jerk and in the wrist accelerometer for jerk cost. Mean jerk and jerk cost increased in the hot condition in the torso and arm accelerometers (Table 3).

**TABLE 3:** Jerk measures measured during a lifting task during the first and fourth bout of work.

Lifting task.						

Mean jerk (units: g/s)						

	Wrist		Torso		Arm	
			
	Hot	Temperate	Hot	Temperate	Hot	Temperate
Circuit 1	2.888 ± 0.594	3.048 ± 0.737	1.361 ± 0.203	1.406 ± 0.304	2.341 ± 0.377	2.499 ± 0.589
Circuit 4	3.561 ± 0.829*	3.145 ± 0.863	1.705 ± 0.404*	1.585 ± 0.308	2.921 ± 0.622*	2.706 ± 0.500

**Maximum jerk (units: g/s)**						

	**Wrist**		**Torso#**		**Arm#**	
			
	**Hot**	**Temperate**	**Hot**	**Temperate**	**Hot**	**Temperate**

Circuit 1	35.21 ± 13.72	32.02 ± 7.49	9.72 ± 3.51	10.58 ± 5.08	18.37 ± 4.75	19.19 ± 4.33
Circuit 4	32.44 ± 10.26	36.65 ± 18.90	13.85 ± 8.96	12.47 ± 6.24	20.63 ± 4.63	24.80 ± 7.78

**Jerk cost (units: g^2^/s)**						

	**Wrist#**		**Torso**		**Arm**	
			
	**Hot**	**Temperate**	**Hot**	**Temperate**	**Hot**	**Temperate**

Circuit 1	2,380 ± 1,140	2,453 ± 971	408 ± 118	438 ± 170	1,263 ± 379	1,411 ± 597
Circuit 4	3,104 ± 1,533	2,939 ± 2,044	619 ± 321*	572 ± 222	1,811 ± 709*	1,721 ± 646

*Data presented as mean ± SD.*

*#A main effect of time was identified in the ANOVA (*p* < 0.05).*

*Circuit 4 different from circuit 1 in that condition (*p* < 0.05).

Subjects displayed a wide range of techniques during the striking and pulling tasks. While the general direction of change in jerk was to increase or stay the same, none of these variables significantly changed during these tasks.

## Discussion

Jerk was increased as measured by accelerometers placed on the wrist, arm, and torso after 40 min of work in a hot environment but typically not different after an identical bout of work in a temperate environment. Paced work performed in hot conditions resulted in higher body temperature and heart rate, and presumably greater fatigue, compared to work performed in temperate conditions. Given the link between fatigue and musculoskeletal injury risk, these data provide preliminary evidence that measuring jerk during physical tasks may provide insights into the objective estimates of injury risk in the workplace.

While the lower extremities contain the primary muscles of gait, core muscles maintain torso position and arm sway varies based on terrain and speed. In the present report, subjects carried a load over one shoulder while walking on a treadmill, which would have resulted in greater torso and arm activation. As such, all three accelerometers measured higher acceleration and jerk during the final circuit. Although we did not place an accelerometer on the lower extremity, similar findings were reported in a previous study of subjects performing simulated occupational tasks while wearing an accelerometer on the ankle (Baghdadi et al., 2018).

During the lifting task, subjects flexed at the hips, knees, and ankles to grasp the object with extended arms and finally bent the elbows to place the object on the table. As such, the arm accelerometer reliably measured higher jerk and acceleration while the wrist accelerometer did so infrequently. The torso-mounted accelerometer measured greater mean jerk and jerk cost at the end of exertion, which is similar to changes reported in a study of normal weight and obese individuals performing a lifting task while wearing an accelerometer on the torso (Ghesmaty Sangachin and Cavuoto, 2016).

The striking task was performed by swinging a long handled dead blow to strike the end of a beam and move it horizontally along a track. Some subjects initiated the swing from the shoulder while other initiated the movement from the waist. Most subjects kept their wrist locked and torso movement was typically minimal, unless the subject was very tall. The pulling task was the most variable in terms of technique, with some subjects moving the torso at the waist and others relying almost exclusively on the arms. This could explain why none of the accelerometers reported a change in acceleration or jerk during these tasks.

Various rehabilitation studies have reported normalized jerk over time and across activities as a means to assess change in impairment (Acuna et al., 2010; Caliguri et al., 2015) and jerk has been used to demonstrate changes in motor performance after therapeutic interventions. Slaboda et al. (2008) found an increase in root-mean-square (RMS) jerk (less smooth) and a less coordinated lifting pattern in a study of lifting kinematics of low back pain patients and healthy controls. Those with low back pain adopted a more cautious lifting strategy. To our knowledge, this is the first study to examine changes in acceleration and jerk in a hot environment and comparing the results to identical work in temperate conditions. Changes in jerk and acceleration were consistently seen in the hot, but not temperate, conditions. We propose that this is due to greater fatigue experienced during work performed in the heat, as indicated by the higher heart rate and body temperature. We hypothesize that similar changes would have been seen in the temperate condition had the subjects performed additional circuits sufficient to result in fatigue.

There are a few limitations that should be noted. Surrogate measures, such as perceived fatigue, and direct measures (e.g., gait changes, decline in maximal strength) were not measured. Vital sign changes demonstrated greater exertion in the hot condition, which we propose is directly related to fatigue. Future studies should correlate changes in jerk to objective measures of fatigue or risk of injury. Failing to specify technique for the included tasks may have resulted in the accelerometers failing to record increases in jerk and acceleration more frequently. Varied technique, however, is common among workers especially between experienced and inexperienced tradesmen (Zhang et al., 2019). Despite varied technique, jerk increased in the hot condition but not in every accelerometer during every task. This suggests that multiple accelerometers may be required for workers with non-repetitive job tasks to identify changes in jerk indicating greater fatigue and risk for injury. The study also was limited to 40 min of exertion, which is shorter than the periods of continuous work that may be experienced in the workplace, particularly for the temperate condition. For work performed in the heat, however, current CDC/NIOSH recommendations suggest workers perform no more than 30 min of sustained medium intensity work per hour at similar temperatures as the conditions tested here (Jacklitsch et al., 2016).

## Conclusion

Forty minutes of paced work performed in the heat resulted in increased jerk in accelerometers placed on the torso, arm, and wrist. The accelerometers most consistently reporting these changes were task specific and suggest that a limited number of worn sensors could identify the onset of fatigue and increased injury risk across an array of occupational duties. Future studies should identify optimal sensor location and correlate changes in body acceleration and jerk with objective measures of fatigue and injury risk.

## Data Availability Statement

The raw data supporting the conclusion of this article will be made available by the authors, without undue reservation.

## Ethics Statement

The studies involving human participants were reviewed and approved by University at Buffalo Institutional Review Board. The patients/participants provided their written informed consent to participate in this study.

## Author Contributions

All authors listed have made a substantial, direct and intellectual contribution to the work, and approved it for publication.

## Conflict of Interest

The authors declare that the research was conducted in the absence of any commercial or financial relationships that could be construed as a potential conflict of interest.

## Publisher’s Note

All claims expressed in this article are solely those of the authors and do not necessarily represent those of their affiliated organizations, or those of the publisher, the editors and the reviewers. Any product that may be evaluated in this article, or claim that may be made by its manufacturer, is not guaranteed or endorsed by the publisher.
